# Virgin coconut oil (VCO) supplementation relieves symptoms and inflammation among COVID-19 positive adults: a single-blind randomized trial

**DOI:** 10.1017/jns.2023.118

**Published:** 2024-01-23

**Authors:** Imelda Angeles-Agdeppa, Jacus S. Nacis, Fabian M. Dayrit, Keith V. Tanda

**Affiliations:** 1 Department of Science and Technology, Food and Nutrition Research Institute, Taguig City, Philippines; 2 Department of Chemistry, Ateneo de Manila University, Quezon City, Philippines

**Keywords:** COVID-19, C-reactive protein (CRP), Symptomatic relief, Virgin coconut oil (VCO)

## Abstract

A clinical study conducted in 2020 showed that virgin coconut oil (VCO) has been found effective in the rapid relief of COVID-19 symptoms and normalization of the C-reactive protein (CRP) concentration among probable and suspected cases of COVID-19. This present study aimed to validate those results and to evaluate the effects of VCO among COVID-19 patients through a 28-day randomized, single-blind trial conducted among 76 SARS-CoV-2 RT-PCR (reverse transcription-polymerase chain report)-confirmed adults, with VCO given as a COVID-19 adjunct therapy. The results showed that VCO recipients were free from symptoms and had normal CRP concentrations by day 14. In comparison, participants in the control group reported relief from signs and symptoms on day 23, with normal CRP concentrations on day 25. This second study bolsters the use of VCO as an effective adjunct therapy for COVID-19-positive patients showing mild-to-moderate symptoms.

## Introduction

The coronavirus disease 2019 (COVID-19) is a highly contagious respiratory disease caused by the SARS-CoV-2 virus. It has resulted in a massive number of deaths worldwide. An important public health strategy against COVID-19 focuses on rapid identification of those who are exposed, quarantine, contract tracing, and early treatment^(1)^. Along with drug discovery initiatives, the inclusion of a diet that can modulate the immune system, proper mental support, and adherence to protocols are being used to manage COVID-19^(2)^ since COVID-19 is shown to be primarily dependent on individual immunity, hence, drugs and traditional medicines with immune-modulating, antiviral, anti-thrombotic, anti-cytokine, and anti-fibrotic properties are being studied for possible use in COVID-19 patients, which has been evident in other studies of adjunct therapies for COVID 19^(3)^, such as Vitamin D^(4)^, melatonin^(5)^, and Vitamin C^(6)^.

A search for natural antiviral compounds revealed that coconut oil possesses antiviral activity against different viruses. A study conducted among paediatric community-acquired pneumonia in the Philippines showed that VCO supplementation of 2 mL/kg/day can aid in the normalization of respiratory rate and resolution of crackles^(7)^. Various animal studies have shown promising effects of VCO supplementation as an adjuvant therapy for chronic allergic lung inflammation and alleviation of airway inflammatory responses^(8,9)^. These beneficial effects can be attributed to the antiviral and anti-inflammatory properties of VCO^(10)^ that come from lauric acid and monolaurin, which are VCO metabolites^(11)^.

Studies have demonstrated that lauric acid and monolaurin can disintegrate the virus envelope in vitro^(12,13)^, inhibit the late stage of the viral replicative cycle^(14)^, and prevent the binding of viral proteins^(15)^, which may lead to the inactivation of viruses such as HIV, measles, herpes simplex-1, vesicular stomatitis, visna, and cytomegalovirus^(16)^. A study has also shown that, in comparison to various lengths of saturated fatty acids (C10:0 to C18:0), lauric acid (C12:0) interferes the most during the replication stage of the Junin virus (JUNV)^(14)^, and VCO is well known to be high in lauric acid. On the other hand, monolaurin can modulate the immune system through the suppression of inflammatory cytokines^(17)^. The immunomodulatory property of monolaurin was implicated in a study conducted among healthcare workers in Italy using untargeted metabolomics, wherein circulating monolaurin level was found to be higher among healthcare workers who were not infected with COVID-19^(18)^. A 2020 dietary intervention conducted in the Philippines, where predetermined doses of VCO were mixed with meals, showed a faster diminishment of symptoms and normalization of CRP levels among those who were given VCO^(19)^. Therefore, this study aims to replicate the 2020 study by investigating the efficacy of VCO against the mutated SARS-CoV-2 virus.

## Materials and methods

### Study design and population

This single-blind randomized controlled trial was conducted at the Valenzuela City Emergency Hospital Philippines from August to November 2021. Seventy-six patients who were COVID-19-positive, exhibiting mild-to-moderate symptoms were enrolled to participate in this study. The classification was based on Memorandum No. 2020-0381 of the Philippines’ Department of Health (DOH), where mild-to-moderate COVID-19 classification is characterized by non-specific symptoms (fever, cough, fatigue, anorexia, myalgia, sore throat, nasal congestion, headache, diarrhoea, nausea, and vomiting), anosmia, ageusia, with or without non-severe pneumonia, and peripheral capillary oxygen saturation (SpO2) of >92%^(20)^. The inclusion criteria were as follows: positive RT-PCR results for COVID-19 (the testing was done within 3 days upon admission), with mild or moderate symptoms, aged 20 years and over, and admitted to the hospital within the past 3 days, controlled hypertension (with maintenance drugs), with normal or slightly elevated AST (aspartate transaminase) and ALT (alanine transaminase) results, and with controlled diabetes. Patients who were vaccinated, pregnant, taking statins, and admitted longer than 3 days were excluded from the study.

The sample size for this study was based on the previous VCO study among suspect and probable cases of COVID-19 in Sta. Rosa City. The calculation utilized the formula for comparing two means, with parameters set at 80% statistical power, a 95% confidence interval (CI), and a standard deviation of 2.6. After accounting for a 10% dropout rate, the final sample size for each group was 40.

All qualified patients were asked for consent before study participation. The study followed the guidelines found in the Declaration of Helsinki and was approved by the Food and Nutrition Research Institute (FNRI) Institutional Review Committee (FIERC-2020-017). The study has also been registered in the Philippine Health Research Registry (PHRR) with the registry ID PHRR220718-004884.

Using an online tool (Research Randomizer^(21)^), the participants were randomly allocated into two groups: the VCO group, which was given a determined dose of VCO, based on their body weight (0.6 to 1.2 mL/kgBW), in a medicinal cup after meals; the control group, which was not given anything aside from the meals and usual medications prescribed by the attending physician. VCO doses were based on a previous study on HIV patients^(22)^. During the initial dose (days 1 to 3), the VCO group was given 0.6 mL/kgBW; succeeding doses were 1.2 mL/kgBW (days 4 to 28)^(19)^.

As a single-blind trial, the research team, including the statistician, was blinded on the type of intervention. The attending physician, focal person, and the project RND kept the unique participant codes until data analysis.

### Data collection

#### Demographic, anthropometric, and biochemical data

Demographic information of participants was collected by the nurses upon admission to the hospital. A calibrated digital weighing scale (SECA® Hamburg, Germany) and stadiometer (SECA® Hamburg, Germany) were used to measure the participant’s weight and height at baseline (day 1), midline (day 14), and endline (day 28). Signs and symptoms are recorded daily, which are fever, cough, fatigue, anorexia, myalgia, sore throat, nasal congestion, headache, diarrhoea, nausea, vomiting, anosmia, and ageusia. Fasting blood sugar(FBS), lipid profile, liver function test (AST/ALT), white blood cell (WBC) count, WBC differential count, and C-reactive protein (CRP) concentration were tested at baseline, midline, and endline. These markers were tested using the 10 mL blood samples that were drawn at baseline, midline, and endline of the study. The blood samples taken for the screening were the same specimen used for the baseline analysis.

The Coulter HmX Analyzer (Beckman Coulter; California, USA) was used for the CBC and WBC analyses. The lipid profile, FBS, and liver function test were analysed using a Unicel DXC 600 Analyzer (Beckman Coulter; California, USA). The Dil-Architect c4000 analyser (Abbott, Germany) was used to determine the CRP concentration. Normal values for CRP are defined as no more than 5 mg/L^(23)^.

#### Dietary data

A calculated 28-day cycle menu (meal recipes) developed by DOST-FNRI was provided to the hospital. These meals were prepared at a central kitchen under the supervision of the hospital registered nutritionist-dietitian (RND) and served to the participants during their stay in the hospital. The meal recipes for the control and the intervention groups were similar, except for the fat source of the intervention, which is the VCO. Corn oil was used for meals requiring oil for the control group (with the restriction on the use of coconut or palm oil). The required energy and nutrients of the participants complied with the recommendation of the European Society of Clinical Nutrition and Metabolism (ESPEN)^(24)^ and the Philippine Department of Health Interim Guidelines^(25)^. There were no dietary restrictions and usual hospital care protocols were observed. Participants’ food intake was recorded daily by the project registered nutritionist-dietitian (RND).

The VCO used for the study was provided by the Philippine Coconut Authority (PCA), and made sure to be compliant with the specified standards of the Philippine National Standard (PNS) (PNS/BAFPS 22:2007), and analysed by the PCA Laboratory Service Division.

The standard values set by PCA are the following: 0.1–0.7% caproic acid, 4–10% caprylic acid, 4–8% capric acid, 45.1–56% lauric acid, 16–21% myristic acid, 7.5–10.2% palmitic acid, 2–5% stearic acid, 5–10% oleic acid, and 1–2.5% linoleic acid.

#### Statistical analysis

The study parameters were assessed on day 1 (baseline), midline (day 14), and endline (day 28). To get the absolute changes from the baseline, different parameters were computed between the 3 measurement periods: baseline vs midline and baseline vs endline. Between the two groups, differences were compared using an unpaired *t*-test, and differences between the periods of measurements were computed within groups. A 0.05 level of significance was used for the test of hypotheses.

Primary outcome analysis was approached as per protocol set. Per protocol set analysis used 75% (i.e. 75% of the 28-day intervention period) compliance to study product intake as compliance to protocol. Only the data from those who consumed 75% of the total VCO were used in the per-protocol analysis of the study. All of the participants included in the analysis have completed the 28-day intervention period.

Descriptive analysis was used to determine the mean and standard error of the anthropometric and biochemical variables. A 2-sided significance level of 5% (p-value<0.05) was used in all analyses to determine whether or not the difference between the two treatment groups was statistically significant.

For qualitative or categorical variables, Pearson’s chi-square test was used to determine the difference between the two groups. Fisher’s exact test was utilized for small cell frequencies. The test of significance for qualitative variables will only be exploratory, and not conclusive.

An independent *t*-test was used to compare the means between VCO and control groups of anthropometric and biochemical variables and the Mann–Whitney U test for non-normal data.

Kaplan–Meier analysis was used to determine differences in the diminishment of COVID-19 symptoms between VCO and control groups.

## Results

### Demographic characteristics of participants

A total of 141 mild and moderate COVID-19 patients were screened. Out of the 141 patients, 61 patients were excluded because of abnormal laboratory results due to impaired lipid profile, high ALT, and FBS. A total of 80 patients qualified as participants were randomly allocated into the VCO group (*n* = 40) and control group (*n* = 40).

**Fig. 1.**
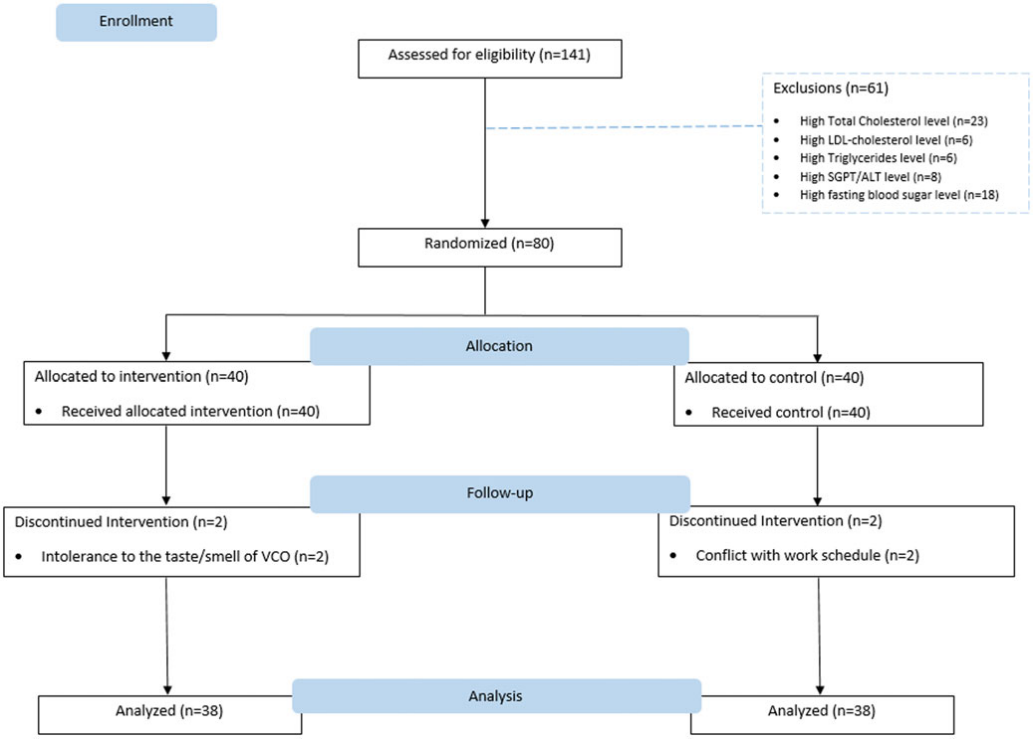
Trial flow diagram. The diagram shows the study’s trial flow and participant allocation and withdrawal.

Seventy-six out of 80 recruited participants completed the 28-day intervention period. Four dropouts in the VCO group reported intolerance to the smell and taste of VCO and two participants in the control group were unable to complete the required follow-up tests. Hence, their data are not included in the presentation of the result (Fig. 1).

Table 1 shows the demographic characteristics of the study participants. The mean age for the VCO and control group was 43.5 and 42.9 years, respectively. In both groups, most of the participants were married (52.6% in VCO and 73.7% in control), had college-level education (44.7% in VCO and 55.3% in control), and employed (84.2% in VCO and 76.3% in control). For body mass index (BMI), 50% of the participants from the VCO group have a normal BMI while 50% of the participants from the control group are overweight/obese.

**Table 1.** Demographic characteristics of participants by group

	Control (*n* = 38)	VCO (*n* = 38)	p-value^1^
	*n* (%)	*n* (%)	
Age, mean ± SD	42.9 ± 15.1	43.5 ± 15.9	0.97
Sex			
Male	24 (63.2)	17 (44.7)	0.13
Female	14 (36.8)	21 (55.3)	
Civil Status			
Single	9 (23.7)	16 (42.1)	0.12
Married	28 (73.7)	20 (52.6)	
Widowed	1 (2.6)	2 (5.3)	
Education Status			
Elementary Level	4 (10.5)	3 (7.9)	0.37
High School Level	7 (18.4)	14 (36.8)	
College Level	21 (55.3)	17 (44.7)	
Vocational Level	6 (15.8)	4 (10.5)	
Employment Status			
Employed	29 (76.3)	32 (84.2)	0.35
Unemployed	9 (23.7)	6 (15.8)	
Number of days stay in hospital, mean ± SD	11.5 ± 4.3	10.5 ± 2.3	0.21
Body Mass Index, *kg/m^2*			
Chronic Energy Deficiency	2 (5.3)	3 (7.9)	0.75
Normal	17 (44.7)	19 (50.0)	
Overweight/Obese	19 (50.0)	16 (42.1)	

1 independent *t*-test, chi-square test, and Fisher’s exact test.

### Diminishing symptoms of participants

Table 2 shows that more than two-thirds of the participants in the VCO group had negative RT-PCR test results (68.4%) by midline compared with 60.5% in the control group. By the end of the intervention, only two participants in the VCO group and three participants in the control group remained positive for COVID-19.

**Table 2.** Results of RT-PCR test by group and period

COVID status	Control (*n* = 38)		VCO (*n* = 38)	
	Positive	Negative	Positive	Negative
	*n* (%)	*n* (%)	*n* (%)	*n* (%)
Baseline	38 (100)	0	38 (100)	0
Midline	15 (39.5)	23 (60.5)	12 (31.6)	26 (68.4)
Endline	3 (7.9)	35 (92.1)	2 (5.3)	36 (94.7)

**Fig. 2.**
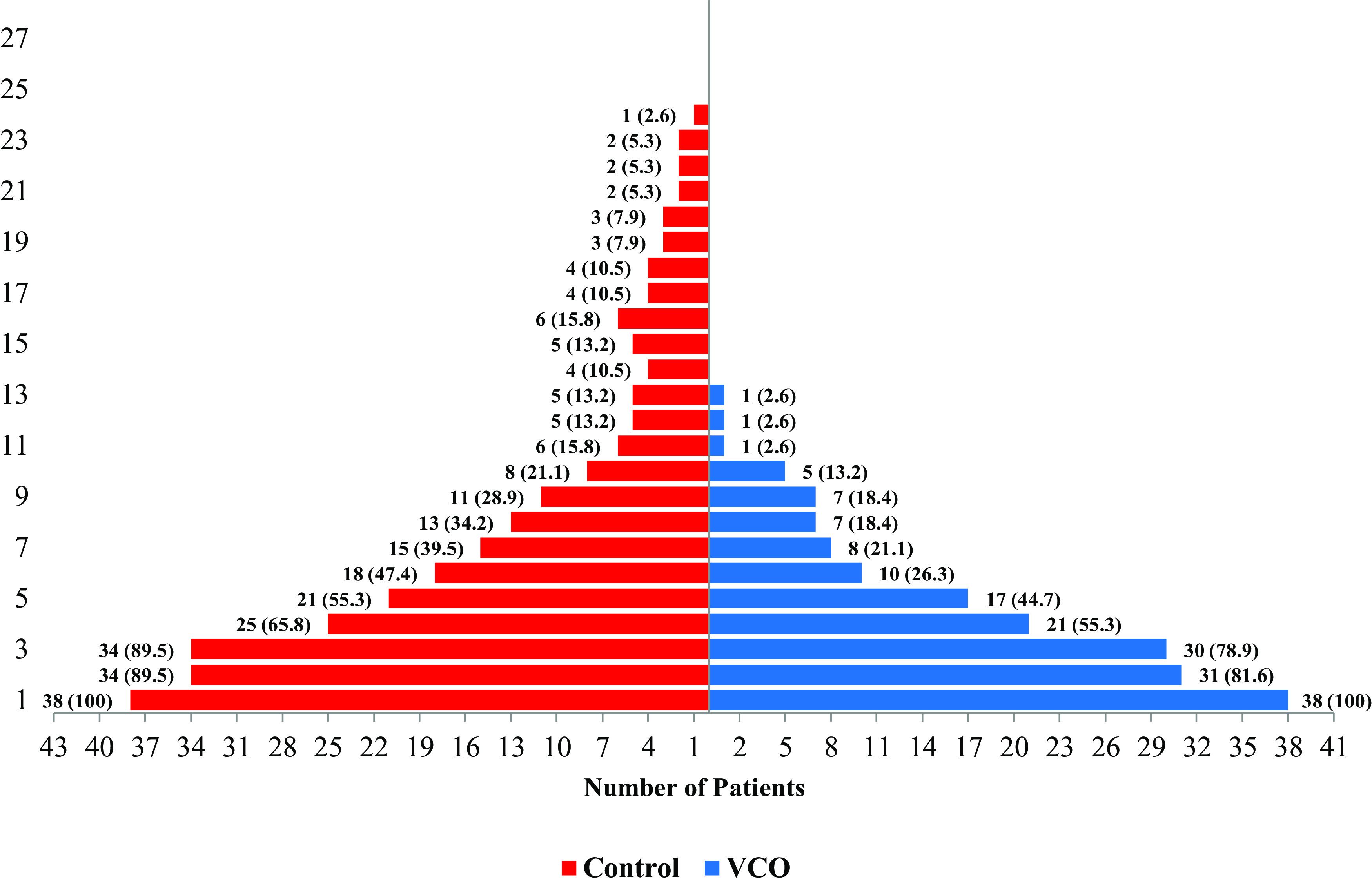
Percentage of participants with diminishing symptoms per group per day. Demonstrates the comparison of signs and symptoms diminishment among VCO and control group.

**Fig. 3.**
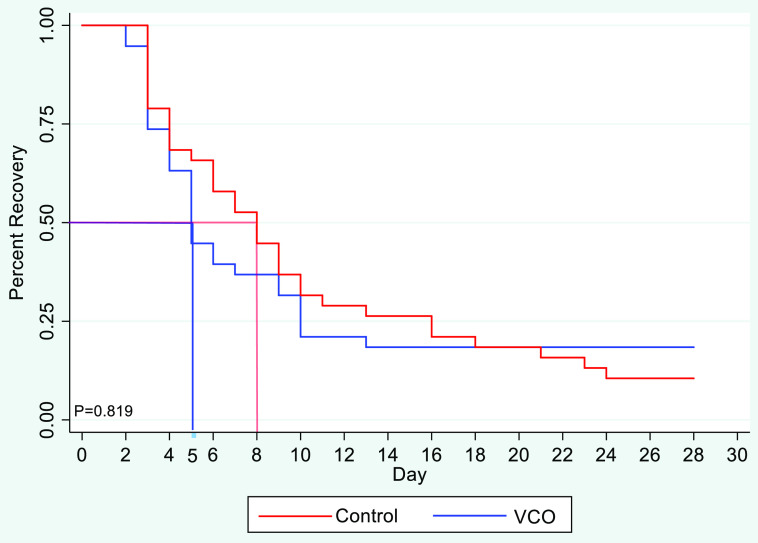
Percentage of recovery from COVID-19 using Kaplan–Meier estimate by VCO and control group. Kaplan–Meier analysis between VCO and control group based on patient recovery.

Figure 2 shows the number of participants with signs and symptoms of COVID-19. At the start of the study, participants from both groups manifested signs and symptoms, which then diminished as the trial progressed. The figure also shows that the symptoms of all participants enrolled in the VCO group had diminished on the 14^th^ day. Meanwhile, participants from the control group still had symptoms until the 24^th^ day – an 11-day difference in terms of the total duration of their signs and symptoms. However, the Kaplan–Meier analysis (Fig. 3) shows that the difference in the relief from symptoms is not significant (*R* = 0.819), although the average time for the diminishment of signs and symptoms of the participants from the VCO group and the control group was 5 and 8 days, respectively.

### Biochemical markers of participants

Table 3 shows that participants in the VCO group were able to demonstrate normal mean CRP concentration (CRP concentration threshold: 5 mg/L) by the midline of the study (day 14). At baseline, the mean CRP concentrations of the VCO and control groups were 8.8 mg/L and 11.6 mg/L, respectively. The CRP concentration of the VCO group fell more rapidly than the control group, crossing the threshold level (5 mg/L) by the midline period (day 14) and falling further to 2.4 at the endline (day 28). In contrast, the control group had a CRP concentration of 10.1 at the midline and improved to 3.3 at the endline. A plot of the CRP trends estimates that the control group fell below the threshold at around day 25.

**Table 3.** Biochemical markers of participants by group and period

	Control (*n* = 38)	VCO (*n* = 38)	p-value^1^
	mean ± SD	mean ± SD	
Fasting Blood Glucose, mg/dL			
Baseline	91.6 ± 11.1	89.7 ± 9.3	0.42
Midline	92.4 ± 12.9	91.8 ± 10.5	0.70
Endline	94.6 ± 11.5	96.6 ± 12.1	0.32
Total Cholesterol, mg/dL			
Baseline	169.5 ± 27.4	161.5 ± 28.6	0.18
Midline	195.6 ± 34.8	214.4 ± 37.4	0.05
Endline	199.9 ± 38.5	213.4 ± 36.8	0.10
LDL-cholesterol, mg/dL			
Baseline	111.1 ± 26.3	97.3 ± 23.9	0.012*
Midline	119.9 ± 33.1	132.0 ± 33.1	0.30
Endline	128.0 ± 35.1	134.1 ± 32.3	0.41
HDL-cholesterol, mg/dL			
Baseline	36.4 ± 9.2	42.6 ± 10.3	0.011*
Midline	42.8 ± 10.8	59.3 ± 12.8	<0.001*
Endline	45.0 ± 9.8	59.1 ± 18.5	0.001*
Triacylglycerol, mg/dL			
Baseline	110.8 ± 39.0	108.2 ± 67.9	0.11
Midline	153.6 ± 92.6	116.4 ± 68.6	0.034*
Endline	135.7 ± 71.8	101.9 ± 46.9	0.05
LDL/HDL, *ratio*			
Baseline	3.3 ± 1.2	2.4 ± 0.9	0.002*
Midline	2.9 ± 1.0	2.3 ± 0.8	0.002*
Endline	3.0 ± 1.0	2.5 ± 0.9	0.018*
TC/HDL, *ratio*			
Baseline	4.9 ± 1.4	4.0 ± 1.1	0.004*
Midline	4.8 ± 1.2	3.8 ± 0.9	0.001*
Endline	4.6 ± 1.3	3.9 ± 1.1	0.005*
Alanine Transaminase, *L*			
Baseline	40.8 ± 23.4	37.1 ± 21.9	0.56
Midline	50.9 ± 34.7	67.4 ± 61.5	0.18
Endline	39.1 ± 24.3	48.7 ± 48.0	0.48
Aspartate Aminotransferase, *L*			
Baseline	35.2 ± 13.9	32.3 ± 16.0	0.15
Midline	33.2 ± 15.5	42.7 ± 31.9	0.33
Endline	30.6 ± 12.8	35.8 ± 20.6	0.44
C-reactive protein, mg/L			
Baseline	11.6 ± 25.4	8.8 ± 16.7	0.81
Midline	10.1 ± 25.5	4.9 ± 8.3	0.95
Endline	3.3 ± 3.3	2.4 ± 2.5	0.29

1 Independent *t*-test and Mann–Whitney U test for non-normal data.

* Significant at p < 0.05.

For FBS, there were no significant differences between the VCO and control groups at baseline, midline, and endline periods of the study. The mean FBS in both groups was still within the normal range (70.10 - 98.93 mg/dL) throughout the intervention.

The study also found that there were significant differences between the VCO and control groups with respect to the lipid parameters, in particular their LDL/HDL and TC/HDL ratios. For the LDL/HDL ratio, the VCO group gave an endline value of 2.5, compared to the control group which had an endline value of 3.0. An LDL/HDL ratio of <2.75 is considered ideal for cardiovascular risk^(26)^. For the TC/HDL ratio, the VCO group gave an endline ratio of 3.9 versus 4.6 for the control group. A TC/HDL ratio of <4.25 is considered ideal for cardiovascular risk^(26)^. The VCO group showed better than the ideal LDL/HDL and TC/HDL ratios throughout the 28-day study period and likewise gave better results than the control group. Conversely, there was no significant difference in the ALT and AST concentration, between the trial arms.

Table 4 shows the distribution of participants by total cholesterol classification and by group and period. At baseline, most of the participants had desirable cholesterol levels in both the VCO (97.4%) and control (86.8%) groups. After the intervention, a change in the total cholesterol status of the VCO group was observed: desirable (29%), borderline high (47.4%), and high (23.7%). The same trend was observed among the participants in the control group. For the triacylglycerol concentration across study periods, most of the participants had desirable triacylglycerol in both VCO (81.6%) and control (86.8%) groups at baseline. After the intervention, the number of participants with desirable triacylglycerol concentration was significantly higher in the VCO group (76.3%) compared to the control (60.5%).

**Table 4.** Distribution of participants by total cholesterol and triacylglycerol classification and by group and period

	Control (*n* = 38)	VC0 (*n* = 38)	p-value
	*n* (%)	*n* (%)	
Total Cholesterol, mg/dL			
Baseline			
Desirable	33 (86.8)	37 (97.4)	0.08
Borderline	5 (13.2)	1 (2.6)	
High	0	0	
Midline			
Desirable	19 (50.0)	13 (34.2)	0.34
Borderline	14 (36.8)	17 (44.7)	
High	5 (13.2)	8 (21.1)	
Endline			
Desirable	17 (44.7)	11 (29.0)	0.19
Borderline	17 (44.7)	18 (47.4)	
High	4 (10.5)	9 (23.7)	
Triacylglycerol, mg/dL			
Baseline			
Desirable	33 (86.8)	31 (81.6)	0.69
Borderline	3 (7.9)	3 (7.9)	
High	2 (5.3)	4 (10.5)	
Midline			
Desirable	23 (60.5)	30 (79.0)	0.3
Borderline	6 (15.8)	3 (7.9)	
High	9 (23.7)	5 (13.2)	
Endline			
Desirable	23 (60.5)	29 (76.3)	0.021*
Borderline	4 (10.5)	7 (18.4)	
High	11 (29.0)	2 (5.3)	

Reference cut-offs: for the total cholesterol: Desirable: <200.00 mg/dL; Borderline high: 200.00–239.38 mg/dL; High: >/ = 239.38 mg/dL; For triacylglycerol: Low: <149.57 mg/dL; Borderline high: 149.57–199.12 mg/dL; High: 200.01–499.14 mg/dL); Very high: >/ = 500.02 mg/dL 1^1^chi-square test and Fisher’s exact test.

* Significant at p < 0.05.

Table 5 shows the distribution of participants by CRP concentration for each group and period. At baseline, more than a third of the participants in the VCO group (34.2%) and control group (39.5%) had CRP concentrations above 5.0 mg/L. After the intervention, the number of participants with CRP concentration above the threshold level was reduced to 13.2% in the VCO group compared with 23.7% in the control group.

**Table 5.** Distribution of participants by CRP concentration for each group and period

	Control (*n* = 38)		VCO (*n* = 38)		p-value^1^
	*n* (%)	*n* (%)	*n* (%)	*n* (%)	
	>5.0	≤ 5.0	>5.0	≤5.0	
C-reactive protein, mg/L					
Baseline	15 (39.5)	23 (60.5)	13 (34.2)	25 (65.8)	0.57
Midline	13 (34.2)	25 (65.8)	8 (21.1)	30 (78.9)	0.17
Endline	9 (23.7)	29 (76.3)	5 (13.2)	33 (86.8)	0.35

1 Chi-square test and Fisher’s exact test.

**Fig. 4.**
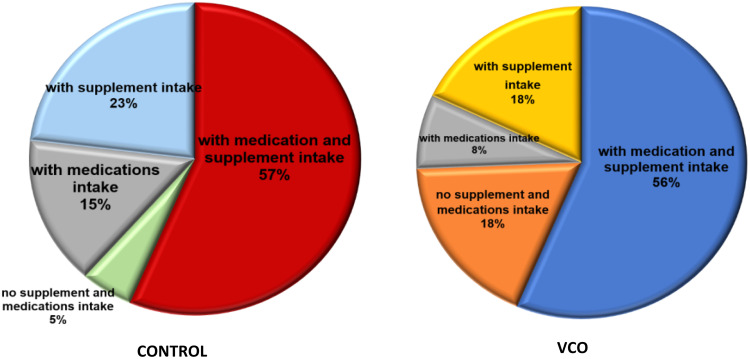
Percentage of medication and supplement intake of participants per group. Demonstrates the percentage of medication and supplement intake per group of participants.

It should also be noted that, unlike in the first study^(19)^, some participants in this study were provided medications and supplements during the trial period. However, more participants in the VCO group (18%) did not take any medications and supplements compared to the control group (5%). The physicians mostly prescribed Omeprazole, Ceftriaxone, Cetirizine, Dexamethasone, and Levofloxacin to the participants during the study (Fig. 4).

## Discussion

Our study demonstrated a faster resolution of COVID-19 symptoms among participants enrolled in the VCO group. Early diminishment of symptoms is beneficial, especially since studies have shown that COVID-19 symptoms can last from 20 days^(27)^ to 83 days^(28)^, which can greatly affect an individual’s quality of life. The anti-inflammatory properties of VCO and its ability to modulate the immune system may have been the main reason behind the early resolution of the participants’ symptoms, especially when fever, which is one of the main symptoms of COVID-19, is a clinical hallmark of inflammation^(10)^. This is also evident with the resolution of anosmia, which is characterized by the decreased, or loss, of an individual’s sense of smell. Anosmia is mainly due to the inflammation of nasal respiratory and olfactory epithelium which results in nasal obstruction^(29)^, hence aroma compounds cannot gain access to the olfactory area.

Anti-inflammatory and antiviral properties of VCO are also exhibited by the early normalization of CRP levels among participants in the VCO Group. CRP is considered an important indicator of infection and inflammation and can be used as an early marker to predict the severity of disease^(30)^. Although CRP concentrations can increase up to 20 to 50 mg/L in patients with severe COVID-19 infection^(31–33)^, mean CRP concentrations for mild and non-severe COVID-19 is at 7.6 mg/dL and above^(30)^, which was also observed in our study.

Starting from a baseline mean CRP concentration of 8.8 ± 16.7 mg/L in the VCO group and 11.6 ± 25.4 mg/L in the control group, it can be inferred that the participants had either an infection or inflammation upon admission. The CRP concentration of participants in the VCO also normalized at day 14, which is 11 days faster than the control group; this result is consistent with the resolution of their COVID-19 symptoms. An earlier study has shown that CRP can be used as a prognostic indicator of COVID-19 among hospitalized patients, as it linearly increases and peaks on the 5^th^ day of infection. The same study also showed that there is a lower peak and earlier decline in the CRP concentrations of the COVID-19 patients who survived than those who died^(34)^, hence, the rapid normalization of CRP concentrations in the VCO group demonstrates that VCO can regulate the inflammatory process effectively. Before the manifestation of symptoms, CRP concentrations will often rise and then eventually drop once the body recovers, making CRP useful in monitoring infections^(35)^. This is consistent with the anti-inflammatory properties of VCO^(36,37)^.

It is also worth noting that despite CRP normalization and symptom resolution of the participants, both the VCO and control groups still had COVID-19-positive participants. This could be due to the prolonged viral shedding of infected individuals^(38,39)^ and possible nucleic acid conversion^(40)^, which then causes a prolonged positive RT-PCR test despite being within the recovery period^(41)^ or fully being recovered^(42)^.

Furthermore, the results of this present study showed that the count for neutrophils, lymphocytes, and eosinophils was normal at baseline until the endline. This is contrary to another study that reported that confined patients with a confirmed COVID-19 diagnosis, had significantly elevated neutrophil, lymphocyte, and eosinophil counts in the biological analysis^(43)^.

The total cholesterol levels of participants increased in both the VCO and control groups, along with this their LDL-cholesterol, and HDL-cholesterol also increased, but were still within the normal range. Triglyceride levels also increased for the control group at the end of the study, all in comparison to their baseline lipid profile results. Such an increase could be attributed to their recovery period from the COVID-19 infection. It has been noted that SARS-CoV-2 viral infection and its pro-inflammatory state negatively impact the lipid metabolism that it has been observed to lower a patient’s LDL and HDL-cholesterol, and increase triglyceride levels upon admission^(44)^ and return to their usual levels 3–6 months after discharge or recovery. This trend has also been associated with the severity of a patient’s symptoms due to possible liver injuries caused by SARS-CoV-2^(45)^, which was reflected in the mean ALT concentrations in both the VCO and control groups. The relationship between the decrease in LDL and the severity of symptoms is also consistent with a study that showed an inverse relationship between CRP and LDL, wherein a high CRP indicates a lower LDL^(45)^. Moreover, the LDL/HDL ratio was lower in the VCO group compared with the control group across periods. Similarly, the mean triacylglycerol concentration of the VCO group throughout the intervention was within the normal range (<149.57 mg/dL) which suggests that the consumption of VCO did not increase the risk for coronary heart disease.

Overall, the VCO group showed a rapid relief from symptoms of COVID-19 and a rapid reduction in mean CRP concentrations compared to the control group. These results are consistent with the findings in the initial study conducted among suspect and probable cases in Sta. Rosa, Laguna, Philippines, wherein the VCO was mixed into their meals with the same dosage used in our study (0.6 to 1.2 mL/kgBW)^(19)^. It should also be noted that the first clinical trial was conducted from July to October 2020 when the predominant variant in the Philippines was the D614G variant^(46)^, while this second clinical trial was conducted from August to November 2021 when the Delta variant was predominant^(47)^.

## Conclusions

This study confirms that VCO aids in the resolution of symptoms and normalization of CRP concentrations among mild-to-moderate cases of COVID-19. Regardless of the modality, whether mixed with meals or taken directly before or after meals, VCO was shown to be effective as an adjunct therapy against COVID-19.

## Strength and limitations

The key strength of this study is its inclusion criteria wherein only participants who were COVID-19 positive and unvaccinated are allowed to partake in the study. Other pertinent COVID-19 biological markers such as D-dimer, IL-6, pulmonary function tests, and chest CT scans were not included as variables in this study, which might have helped establish the utility of VCO. Furthermore, the research was implemented at the peak of the Philippines’ vaccination drive, thus, limiting the qualified participants even further.
